# Dosimetric impact of calibration coefficients determined using linear accelerator photon and electron beams for ionization chamber in an on-site dosimetry audit

**DOI:** 10.1093/jrr/rrae054

**Published:** 2024-08-17

**Authors:** Kensuke Tani, Akihisa Wakita, Naoki Tohyama, Yukio Fujita

**Affiliations:** Division of Medical Physics, EuroMediTech Co., Ltd, 2-20-4 Higashi-Gotanda, Shinagawa, Tokyo 141-0022, Japan; Division of Medical Physics, EuroMediTech Co., Ltd, 2-20-4 Higashi-Gotanda, Shinagawa, Tokyo 141-0022, Japan; Department of Health Sciences, Komazawa University, 1-23-1 Komazawa, Setagaya, Tokyo 154-8525, Japan; Department of Health Sciences, Komazawa University, 1-23-1 Komazawa, Setagaya, Tokyo 154-8525, Japan

**Keywords:** audit, calibration coefficient, ionization chamber, plastic phantom

## Abstract

This study aimed to clarify the dosimetric impact of calibration beam quality for calibration coefficients of the absorbed dose to water for an ionization chamber in an on-site dosimetry audit. Institution-measured doses of 200 photon and 184 electron beams were compared with the measured dose using one year data before and after the calibration of the ionization chamber used. For photon and electron reference dosimetry, the agreements of the institution-measured dose against two measured doses in this audit were evaluated using the calibration coefficients determined using ^60^Co (${N}_{D,\mathrm{w},{}^{60}\mathrm{Co}}$) and linear accelerator (linac) (${N}_{D,\mathrm{w},Q}$) beams. For electron reference dosimetry, the agreement of two institution-measured doses against the measured dose was evaluated using${N}_{D,\mathrm{w},Q}$. Institution-measured doses were evaluated using direct- and cross-calibration coefficients. For photon reference dosimetry, the mean differences and standard deviation (SD) of institution-measured dose against the measured dose using ${N}_{D,\mathrm{w},{}^{60}\mathrm{Co}}$ and ${N}_{D,\mathrm{w},Q}$ were −0.1% ± 0.4% and −0.3% ± 0.4%, respectively. For electron reference dosimetry, the mean differences and SD of institution-measured dose using the direct-calibration coefficient against the measured dose using ${N}_{D,\mathrm{w},{}^{60}\mathrm{Co}}$ and ${N}_{D,\mathrm{w},Q}$ were 1.3% ± 0.8% and 0.8% ± 0.8%, respectively. Further, the mean differences and SD of institution-measured dose using the cross-calibration coefficient against the measured dose using ${N}_{D,\mathrm{w},Q}$ were −0.1% ± 0.6%. For photon beams, the dosimetric impact of introducing calibration coefficients determined using linac beams was small. For electron beams, it was larger, and the measured dose using ${N}_{D,\mathrm{w},Q}$ was most consistent with the institution-measured dose, which was evaluated using a cross-calibration coefficient.

## INTRODUCTION

The accuracy of the dose delivered from a linear accelerator (linac) is one of the most important and fundamental quality control parameters in radiation therapy [1–5]. Reference dosimetry is a starting point for ensuring the quality of dose distributions delivered to patients. Radiation therapy institutions control the reference dose output from linac within 1–2% [4, 5] through reference dosimetry following each standard protocol [6–11]. The International Atomic Energy Agency (IAEA) indicated that, from a radiobiological point of view, it is reasonable to maintain the systematic bias of the reference dose within 1–2% [1, 2].

Variations in the reference dose could directly affect the quality of the delivered dose distributions in all patients, and various factors may contribute to this variation [12]. Therefore, it is recognized that quality assurance of the reference dose not only requires control by the institution itself, but also checks via an individual dosimetry audit [1–3, 12–19]. Several organizations, such as the IAEA [20–24], Imaging and Radiation Oncology Core (IROC) [12, 25, 26] and National Physical Laboratory [16], have established individual dosimetry audits and reported their usefulness. Through the intervention of these audits, variation in the reference dose in clinical institutions has decreased over the years [12, 16, 24], making significant contributions to medical safety.

However, there are limited methods for institutions to objectively confirm whether their reference dosimetry is within ±2%. Although individual dosimetry audits might be a way to confirm it, most individual dosimetry audits, such as the IAEA [20–24], IROC [12, 26, 27], European Organization for Research and Treatment of Cancer [27], Japan Clinical Oncology Group [28] and Radiation Dosimetry Service [29], set a large tolerance of 5% for the agreement between their measured dose and institution-measured dose [12]. While the tolerance is reasonably set due to their remoteness and wider contributions, it would be better if some audits that could operate with a tighter tolerance, such as 1–2% [4, 5], and meet the recommendations [1, 2] and guidelines [4, 5] are available.

**Fig. 1:**
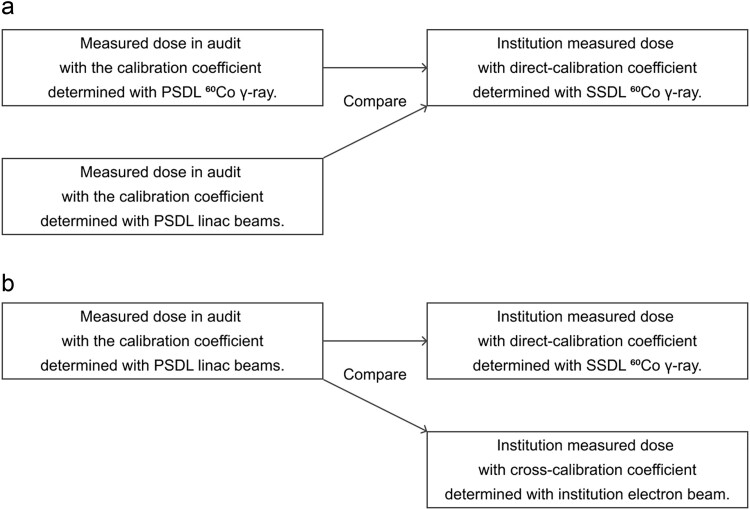
Overview of comparisons of institution-measured dose against the measured dose in this audit for photon and electron reference dosimetry (a) and additional comparisons for electron reference dosimetry (b). (a) For photon and electron reference dosimetry, investigated for the calibration coefficients in this audit. (b) For electron reference dosimetry, investigated for the calibration coefficients in institutions. PSDL, primary standard dosimetry laboratory; SSDL, secondary standard dosimetry laboratory.

The purpose of this study was to clarify the dosimetric impact of calibration beam quality for calibration coefficients of the absorbed dose to water for an ionization chamber in an on-site dosimetry audit and to establish a more reliable practice for dosimetry audit. Furthermore, the uncertainty in this dosimetry audit was evaluated, and appropriate calibration coefficients and tolerances were discussed based on the dosimetry audit results. This on-site dosimetry audit was updated with the related parameters from our previous work [30] and the ionization chamber used in this audit was calibrated with ^60^Co γ-ray and linac photon and electron beams in a primary standard dosimetry laboratory (PSDL). An overview of this study is shown in Fig. 1, which shows two comparisons of the institution-measured doses against the measured dose in this audit. For photon and electron reference dosimetry, the institution-measured dose was compared with the measured dose in this audit using the calibration coefficients at beam quality ^60^Co (${N}_{D,\mathrm{w},{}^{60}\mathrm{Co}}$) and Q of the linac beams (${N}_{D,\mathrm{w},Q}$). Furthermore, for electron reference dosimetry, the measured dose in this audit was compared with the institution-measured dose using two types of calibration coefficients: (i) the direct-calibration coefficient, which was determined with ^60^Co γ-ray in secondary standard dosimetry laboratory (SSDL) and (ii) the cross-calibration coefficients, which was determined using institution’s maximum energy of electron beams with reference to the institution’s Farmer chamber calibrated with SSDL ^60^Co γ-ray. Based on these results, the dosimetric impact of the calibration coefficients in this dosimetry audit is discussed.

## MATERIALS AND METHODS

### Audit system for photon reference dosimetry

**Fig. 2:**
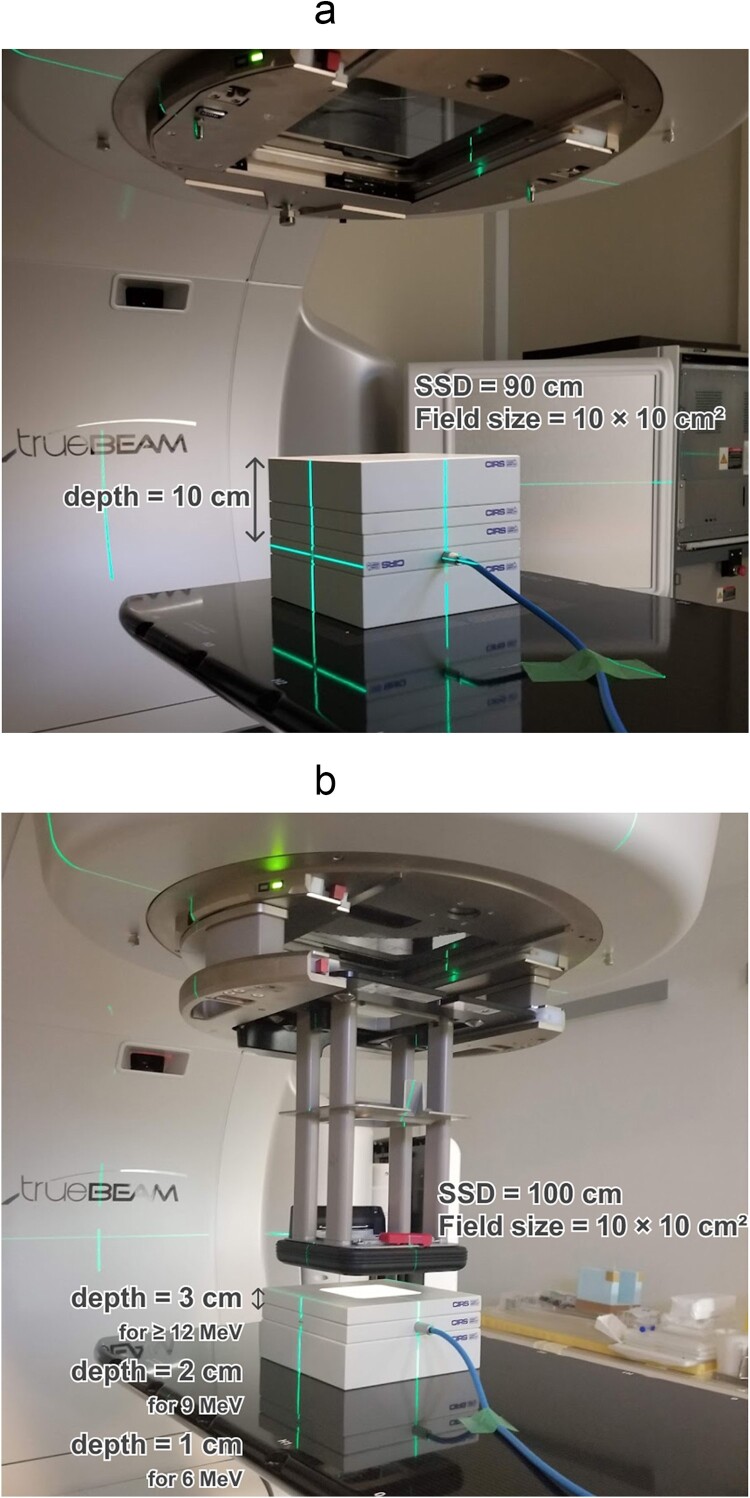
Appearance of the dosimetry audit measurement for (a) photon and (b) electron reference dosimetry. (a) Audit for photon reference dosimetry. (b) Audit for electron reference dosimetry.

This audit system was an on-site dosimetry audit using a Farmer-type ionization chamber (30013, PTW, Freiburg, Germany), an electrometer (UNIDOSwebline, PTW), and a plastic phantom (Plastic Water Diagnostic-Therapy; PWDT, CIRS, Norfolk, VA, USA). The appearance of this dosimetry audit for photon reference dosimetry is shown in Fig. 2a. The Farmer chamber was set at a depth of 10 cm and source-to-chamber distance of 100 cm in the plastic phantom. The Farmer chamber was calibrated with γ-ray of ^60^Co and X-ray (6, 10 and 15 MV) of the linac in PSDL. The absorbed dose to water ${D}_{\mathrm{w},Q}$ was evaluated using the following equations:

(with the calibration coefficient of the absorbed dose to water at the beam quality ^60^Co γ-ray:${N}_{D,\mathrm{w},{}^{60}\mathrm{Co}}$)


(1) \begin{equation*} {D}_{\mathrm{w},Q}=M\ {N}_{D,\mathrm{w},{}^{60}\mathrm{Co}}\ {k}_{Q,{}^{60}\mathrm{Co}}\ {k}_{\mathrm{pl},Q} \end{equation*}

(with the calibration coefficient of the absorbed dose to water at beam quality Q of the linac beams:${N}_{D,\mathrm{w},Q}$)


(2) \begin{equation*} {D}_{\mathrm{w},Q}=M\ {N}_{D,\mathrm{w},Q}\ {k}_{\mathrm{pl},Q} \end{equation*}

where *M* is the fully corrected electrometer reading in coulombs (C), which was corrected for ion recombination (*k*_s_), polarity effect (*k*_pol_), environmental conditions (temperature and pressure, *k*_TP_) and electrometer calibration factor (*k*_elec_). ${N}_{D,\mathrm{w},{}^{60}\mathrm{Co}}$ and ${N}_{D,\mathrm{w},Q}$ are the calibration coefficients of absorbed dose to water at the beam quality ^60^Co γ-ray and *Q* of linac beams. ${k}_{Q,{}^{60}\mathrm{Co}}$ is the beam quality conversion factor from the beam quality ^60^Co. ${k}_{\mathrm{pl},Q}$ is the fluence scaling factor for the ionization chamber and plastic phantom material.

*k*_s_ were measured at the first 16 institutions and the average value was adopted for each beam quality. *TPR*_20,10_ to calculate ${k}_{Q,{}^{60}\mathrm{Co}}$ and ${N}_{D,\mathrm{w},Q}$ were collected at the first 20 institutions for the TrueBeam linac (Varian Medical Systems, Palo Alto, CA, USA), and the average value was adopted for each beam quality. ${k}_{Q,{}^{60}\mathrm{Co}}$ referred a dosimetry protocol published by the Japanese Society of Medical Physics (JSMP12) [11]. *k*_pol_ was determined to be 1.000 for all beam qualities [11, 31]. The depth-scaling factor (*C*_pl_) of the PWDT was determined to be 1.000 because of its relative electron density (g/cm^3^) of 1.003 [32]. ${k}_{\mathrm{pl},Q}$ was determined from the ratio of the measured dose to water in water and plastic phantom at an institution. ${D}_{\mathrm{w},Q}$ for the flattening-filter-free (FFF) beams was further corrected using the volume averaging correction factor (*k*_vol_) and the ratios of water to air stopping-power ratios for the FFF and weighted flattening-filter (WFF) beams ${({s}_{\mathrm{w},\mathrm{air}})}_{\mathrm{WFF}}^{\mathrm{FFF}}$ by following IAEA TRS-483 [10].

All the parameters are shown in Table 1.

**Table 1:** Parameters to evaluate the absorbed dose to water in the dosimetry audit for photon reference dosimetry

X-ray energy	4 MV	6 MV	8 MV	10 MV	15 MV	6 MV FFF	10 MV FFF
*TPR*_20,10_	0.621	0.667	0.710	0.739	0.764	0.631	0.706
*k*_s_	1.002	1.003	1.003	1.004	1.006	1.006	1.010
*k*_pol_	1.000	1.000	1.000	1.000	1.000	1.000	1.000
*k*_pl_	0.998	0.996	0.995	0.996	0.996	0.997	0.996
*k*_vol_						1.002	1.005
${({s}_{\mathrm{w},\mathrm{air}})}_{\mathrm{WFF}}^{\mathrm{FFF}}$						0.999	0.996

TPR: Tissue-phantom ratio, FFF: flattening-filter-free, WFF: weighted flattening-filter, *k*_s_: ion recombination correction factor, *k*_pol_: polarity effect correction factor, *k*_pl_: fluence scaling factor, *k*_vol_: volume averaging correction factor, ${\left({s}_{w,\mathrm{air}}\right)}_{\mathrm{WFF}}^{\mathrm{FFF}}$: water-to-air stopping-power ratios for the FFF and WFF beams.

The ${D}_{\mathrm{w},Q}$ in this audit, evaluated using Equations (1) and (2), was compared at a depth of 10 cm with the institution-measured dose obtained in the last three days. The institutions in this study used the ionization chambers of PTW 30013, IBA FC65-G (IBA Dosimetry, Schwarzenbruck, Germany), IBA FC23-C and Exradin A12 (Standard Imaging, Middleton, WI, USA) with a dosimetry protocol of the JSMP12 [11] for photon reference dosimetry. These institution ionization chambers were calibrated with ^60^Co γ-ray in SSDL. The audit results in this study summarized the one year data before and after the calibration of the ionization chamber used in this audit (from November 2021 to October 2023). The number and type of linacs in this study were 38 TrueBeam/VitalBeam (Varian), four Clinac (Varian), three Halcyon/Ethos (Varian) and one VersaHD (Elekta Oncology Systems, Crawley, UK). A total of 200 photon beams (WFF: 123 beams / FFF: 77 beams) were evaluated.

### Audit system for electron reference dosimetry

The appearance of the dosimetry audit for electron reference dosimetry is shown in Fig. 2b. The Farmer chamber was set at a depth of 1, 2 and 3 cm for 6, 9 and ≥12 MeV, respectively, with a source-to-surface distance (SSD) of 100 cm in the plastic phantom. The Farmer chamber was calibrated with γ-ray of ^60^Co and electron beam (9, 12, 15 and 18 MeV) of the linac in PSDL. The absorbed dose to water at the depth of maximum ${D}_{\mathrm{w},Q}\left({d}_{\mathrm{max}}\right)$ was evaluated using the following equations:

(with the calibration coefficient of the absorbed dose to water at the beam quality ^60^Co γ-ray:${N}_{D,\mathrm{w},{}^{60}\mathrm{Co}}$)


(3) \begin{align*} {D}_{\mathrm{w},Q}({d}_{\mathrm{m}\mathrm{ax}})=&M({d}_{\mathrm{m}}){N}_{D,\mathrm{w},{}^{60}\mathrm{Co}}\ {k}_{Q,{}^{60}\mathrm{Co}}\ {k}_{\mathrm{pl},Q}\ {({(\overline{L}/\rho )}_{\mathrm{w},\mathrm{air}})}_{d_{\mathrm{m}},{d}_{\mathrm{c}}}\nonumber\\& {P}_{d_{\mathrm{m}},{d}_{\mathrm{c}}}/ PDD({d}_{\mathrm{m}}) \end{align*}

(with the calibration coefficient of the absorbed dose to water at beam quality Q of the linac beams:${N}_{D,\mathrm{w},Q}$)


(4) \begin{align*} {D}_{\mathrm{w},Q}({d}_{\mathrm{m}\mathrm{ax}})=&M({d}_{\mathrm{m}}){N}_{D,\mathrm{w},Q}\ {k}_{\mathrm{pl},Q}\ {({(\overline{L}/\rho )}_{\mathrm{w},\mathrm{air}})}_{d_{\mathrm{m}},{d}_{\mathrm{c}}}\nonumber\\ &{P}_{d_{\mathrm{m}},{d}_{\mathrm{c}}}/ PDD({d}_{\mathrm{m}}) \end{align*}

A half-value depth (*R*_50_) was determined from the average value of the *R*_50_ of the percentage depth doses (PDDs) measured using a Roos (34001, PTW) and an Advanced Markus (34045, PTW), while *PDD*(*d*_m_) was determined from the *PDD*s measured using a microDiamond detector (60019, PTW), using a BeamScan 3D water phantom (PTW) at an institution (Varian TrueBeam), and the value was adopted for each beam quality. ${k}_{Q,{}^{60}\mathrm{Co}}$ of the PTW30013 chamber for the electron beams was based on a previously reported value [33]. The physical density and *C*_pl_ of the PWDT were 1.039 [32] and 0.959 [32], respectively; therefore, the depth of measurement was not corrected. ${k}_{\mathrm{pl},Q}$ was determined from measurements. ${({(\overline{L}/\rho )}_{\mathrm{w},\mathrm{air}})}_{d_{\mathrm{m}},{d}_{\mathrm{c}}}$ were the ratios of water to air stopping-power ratios for a depth of measurement (*d*_m_) and reference depth (*d*_c_) and calculated according to the JSMP12 [11]. ${P}_{d_{\mathrm{m}},{d}_{\mathrm{c}}}$ are the ratios of the perturbation correction factors for the PTW30013 chamber at the depth of measurement and reference depth, and were determined from the measurements. All parameters are listed in Table 2.

**Table 2:** Parameters to evaluate the absorbed dose to water in the dosimetry audit for electron reference dosimetry

Electron energy	6 MeV	9 MeV	12 MeV	15 MeV	16 MeV	18 MeV	20 MeV
*d*_m_ (cm)	1.0	2.0	3.0	3.0	3.0	3.0	3.0
*R*_50_ (cm)	2.40	3.63	5.07	6.37	6.69	7.59	8.29
${({(\overline{L}/\rho )}_{\mathrm{w},\mathrm{air}})}_{d_{\mathrm{m}},{d}_{\mathrm{c}}}$	0.990	0.998	1.001	0.988	0.985	0.978	0.974
*PDD* (*d*_m_)	0.958	0.997	1.000	0.999	1.000	0.999	0.996
*k*_s_	1.009	1.009	1.009	1.009	1.009	1.009	1.010
*k*_pol_	1.000	1.000	1.000	1.000	1.000	1.000	1.000
*k*_pl_	1.006	1.002	1.002	1.002	1.002	1.001	1.001
${P}_{d_{\mathrm{m}},{d}_{\mathrm{c}}}$	1.008	1.001	0.999	1.007	1.008	1.012	1.010

*d*_m_: depth of measurement, *R*_50_: half-value depth, ${({(\overline{L}/\rho)}_{w,\mathrm{air}})}_{d_m,{d}_c}$: ratios of water to air stopping-power ratios for a depth of measurement and reference depth, PDD: percentage depth doses, *k*_s_: ion recombination correction factor, *k*_pol_: polarity effect correction factor, *k*_pl_: fluence scaling factor, ${P}_{d_m,{d}_c}$: ratios of the perturbation correction factors for the PTW30013 chamber at the depth of measurement and reference depth.

The ${D}_{\mathrm{w},Q}\left({d}_{\mathrm{max}}\right)$ in this audit, evaluated using Equations (3) and (4), was compared at a depth of maximum with the institutional-measured dose obtained in the last three days. The institutions in this study used ionization chambers PTW Roos (34001), PTW Advanced Markus (34045), IBA NACP-02 and IBA PPC40 using the dosimetry protocol by the JSMP12 for electron reference dosimetry. These institution ionization chambers were calibrated with ^60^Co γ-ray in SSDL. Furthermore, the institutions reported, if available, two kinds of ${D}_{\mathrm{w},Q}\left({d}_{\mathrm{max}}\right)$ which were evaluated with direct- and cross-calibration coefficients with related parameters such as ${k}_{Q,{}^{60}\mathrm{Co}}$ and ${k}_{Q,{Q}_{cross}}$ according to the JSMP12 [11]. The number and type of linacs used in this study were 37 TrueBeam/VitalBeam (Varian), four Clinac (Varian) and one VersaHD (Elekta). A total of 184 electron beams were evaluated (with direct-calibration coefficients: 184 beams / with cross-calibration coefficients: 76 beams).

## RESULTS

### Audit results for photon reference dosimetry

**Fig. 3:**
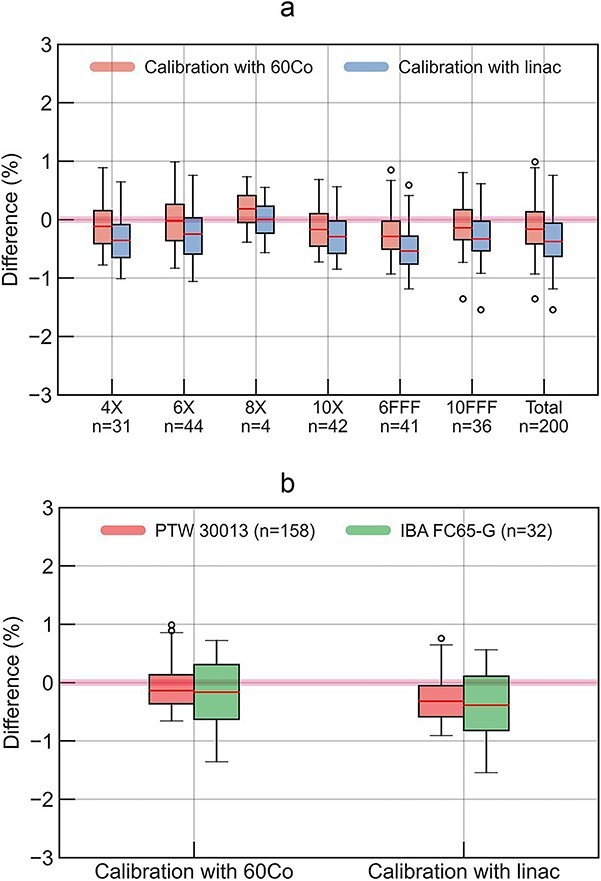
For photon reference dosimetry, the differences of institution-measured dose against two types of the measured dose in this audit evaluated using the calibration coefficients determined using ^60^Co and linac beams. (a) and (b) show the agreements for each X-ray energy and ionization chamber. (a) X-ray energy, (b) Chamber type.

Figure 3 shows box plots for the differences of the institution-measured dose against the measured dose in this audit using ${N}_{D,\mathrm{w},{}^{60}\mathrm{Co}}$ and ${N}_{D,\mathrm{w},Q}$ for photon reference dosimetry. For total evaluation shown in Fig. 3a and b, the mean differences and standard deviation (SD) using ${N}_{D,\mathrm{w},{}^{60}\mathrm{Co}}$ were − 0.1% ± 0.4%, while those using ${N}_{D,\mathrm{w},Q}$ were − 0.3% ± 0.4%. The mean differences and SD using ${N}_{D,\mathrm{w},{}^{60}\mathrm{Co}}$ were − 0.1% ± 0.4% for the institutions using PTW 30013 and − 0.2% ± 0.5% for those using IBA FC65-G, whereas the mean differences and SD using ${N}_{D,\mathrm{w},Q}$ were − 0.3% ± 0.4% for PTW 30013 and − 0.4% ± 0.5% for IBA FC65-G. The differences in the measured doses in this audit between the use of ${N}_{D,\mathrm{w},{}^{60}\mathrm{Co}}$ and ${N}_{D,\mathrm{w},Q}$ were less than 0.3% for all photon energy in this study. Since only two cases were performed for 15 MV X-ray, they were included in the total evaluation but not shown as box plots in Fig. 3a.

### Audit results for electron reference dosimetry

**Fig. 4:**
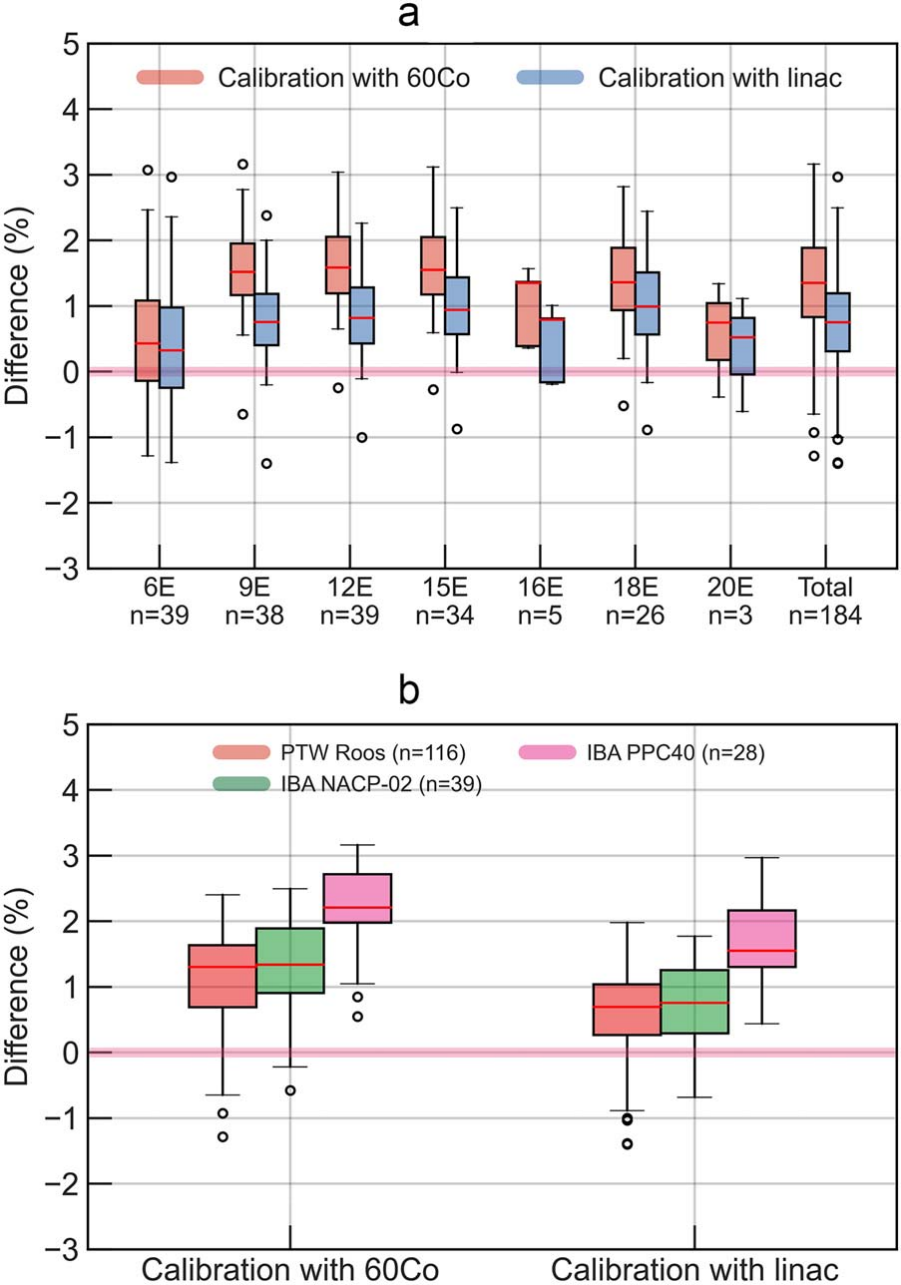
For electron reference dosimetry, the differences of institution-measured doses against two types of the measured dose in this audit evaluated using the calibration coefficients determined using ^60^Co and linac beams. The institution-measured doses were evaluated using direct-calibration coefficient. (a) and (b) show the agreements for each electron energy and ionization chamber. (a) Electron energy. (b) Chamber type.

Figure 4 shows box plots for the differences of institutional-measured dose from the measured dose in this audit for electron reference dosimetry. For total evaluation shown in Fig. 4a and b, the mean differences and SD using ${N}_{D,\mathrm{w},{}^{60}\mathrm{Co}}$ were 1.3% ± 0.8%, while those using ${N}_{D,\mathrm{w},Q}$ were 0.8% ± 0.8%. The mean differences and SD using ${N}_{D,\mathrm{w},{}^{60}\mathrm{Co}}$ were 1.1% ± 0.8%, 1.3% ± 0.7% and 2.2% ± 0.7% for the institutions using PTW Roos, IBA NACP-02 and IBA PPC40, respectively, whereas the mean differences and SD using ${N}_{D,\mathrm{w},Q}$ were 0.6% ± 0.7% for PTW Roos, 0.8% ± 0.6% for IBA NACP-02 and 1.6% ± 0.6% for IBA PPC40. When ${N}_{D,\mathrm{w},{}^{60}\mathrm{Co}}$ and ${N}_{D,\mathrm{w},Q}$ were used, the measured doses in this audit differed by 0.1%, 0.8%, 0.8%, 0.6%, 0.6%, 0.4% and 0.2% for the 6, 9, 12, 15, 16, 18 and 20 MeV electron beams, respectively.

**Fig. 5:**
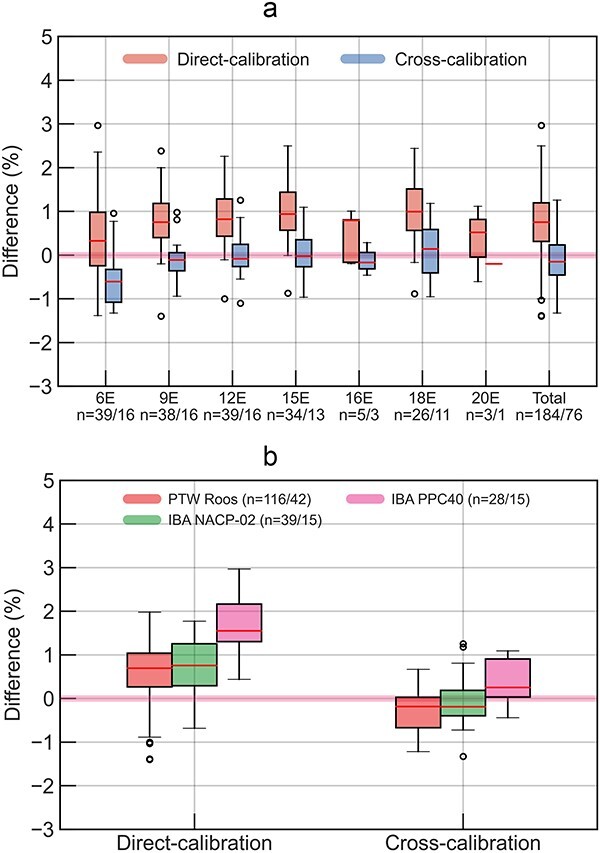
For electron reference dosimetry, the agreement of two types of institution-measured doses against the measured dose in this audit evaluated using the calibration coefficients determined using linac beams. The institution-measured doses were evaluated using direct- and cross-calibration coefficients. (a) and (b) show the agreements for each electron energy and ionization chamber. (a) Electron energy, (b) chamber type.

Figure 5 shows box plots for the agreement of institution-measured doses evaluated using direct and cross-calibration coefficients against the measured dose in this audit using ${N}_{D,\mathrm{w},Q}$ for electron reference dosimetry. For total evaluation, the mean differences and SD using direct- and cross-calibration coefficient were 0.8% ± 0.8% and − 0.1% ± 0.6%, respectively. The agreements were the closest when institutions adopted a cross-calibration coefficient, and this audit adopted ${N}_{D,\mathrm{w},Q}$ in this study.

### Uncertainty for the measured absorbed dose to water in this audit

Uncertainties for the measured absorbed dose to water in this audit for photon and electron beams using the calibration coefficients determined using ^60^Co γ-ray and linac beams were estimated and are summarized in Tables 3-5. The expanded relative standard uncertainty (k = 2) for the measured absorbed dose to water of the WFF photon beams in this audit was 1.4%, as shown in Table 3. The expanded relative standard uncertainty (k = 2) for the measured absorbed dose to water of the FFF photon beams in this audit was 1.5%, as shown in Table 4. The expanded relative standard uncertainty (k = 2) for the measured absorbed dose to water of the electron beams in this audit was 1.9%, as shown in Table 5. Individual differences of plastic phantom shown in Tables 3 and 5 as a component of uncertainty include the individual differences in thickness, nominal physical density, and nominal relative electron density.

**Table 3:** Uncertainty budgets for the measured absorbed dose to water of the WFF photon beams in this audit

Component of uncertainty	Calibration with ^60^Co	Calibration with linac
Calibration coefficient ${N}_{D,\mathrm{w},{}^{60}\mathrm{Co}}$	0.4%^11)^	
Beam quality conversion factor ${k}_{Q,{}^{60}\mathrm{Co}}$	1.0%^11)^	
Assignment of ${k}_{Q,{}^{60}\mathrm{Co}}$	0.1%	
Calibration coefficient ${N}_{D,\mathrm{w},Q}$		0.5%
Assignment of ${N}_{D,\mathrm{w},Q}$		0.1%
Long term chamber stability	0.2%	0.2%
Measurement setting	0.2%	0.2%
Correction factor for measured value *k*_i_	0.2%	0.2%
Fluence scaling factor *k*_pl_	0.3%	0.3%
Individual difference of plastic phantom	0.2%	0.2%
Measurement value *M*_raw_	0.1%	0.1%
Combined relative standard uncertainty	1.2%	0.7%
Expanded relative standard uncertainty (*k* = 2)	2.4%	1.4%

**Table 4:** Uncertainty budgets for the measured absorbed dose to water of the FFF photon beams in this audit

Component of uncertainty	Calibration with ^60^Co	Calibration with linac
Uncertainty for WFF photon dosimetry	1.2%	0.7%
Stopping power ratio (*s*_w,air_)_FFF,WFF_	0.2%^10)^	0.2%^10)^
Correction for volume averaging effect *k*_vol,Q_	0.2%^10)^	0.2%^10)^
Combined relative standard uncertainty	1.2%	0.8%
Expanded relative standard uncertainty (*k* = 2)	2.5%	1.5%

**Table 5:** Uncertainty budgets for the measured absorbed dose to water of the electron beams in this audit

Component of uncertainty	Calibration with ^60^Co	Calibration with linac
Calibration coefficient ${N}_{D,\mathrm{w},{}^{60}\mathrm{Co}}$	0.4%^11)^	
Beam quality conversion factor ${k}_{Q,{}^{60}\mathrm{Co}}$	0.2%^33)^	
Assignment of ${k}_{Q,{}^{60}\mathrm{Co}}$	0.1%	
Calibration coefficient ${N}_{D,\mathrm{w},Q}$		0.6%
Assignment of ${N}_{D,\mathrm{w},Q}$		0.1%
Long term chamber stability	0.2%	0.2%
Measurement setting	0.2%	0.2%
Correction factor for measured value *k*_i_	0.2%	0.2%
Fluence scaling factor *k*_pl_	0.3%	0.3%
Individual difference of plastic phantom	0.2%	0.2%
Measurement value *M*_raw_	0.1%	0.1%
Perturbation correction factor ratio of measurement depth to calibration depth ${P}_{d_{\mathrm{m}},{d}_{\mathrm{c}}}$	0.3%	0.3%
Stopping power ratio of measurement depth to calibration depth ${({(\overline{L}/\rho )}_{\mathrm{w},\mathrm{air}})}_{d_{\mathrm{m}},{d}_{\mathrm{c}}}$	0.3%	0.3%
*PDD* at measurement depth *PDD* (*d*_m_)	0.3%	0.3%
Combined relative standard uncertainty	0.9%	0.9%
Expanded relative standard uncertainty (*k* = 2)	1.7%	1.9%

## DISCUSSION

Currently, ^60^Co-based protocols are widely used for reference dosimetry, and the dosimetric impact and benefit on introducing the calibration coefficients determined using linac beams instead of ^60^Co γ-ray has not been well clarified [34]. However, the results of this on-site dosimetry audit revealed a dosimetric impact. The impact for the photon beams on this dosimetry audit results was small. Regardless of whether the measured dose in this audit was evaluated with ${N}_{D,\mathrm{w},{}^{60}\mathrm{Co}}$ or ${N}_{D,\mathrm{w},Q}$, both of the agreements between the measured dose in this audit and institutions were almost same and within ±1.5%. This indicates that the ${k}_{Q,{}^{60}\mathrm{Co}}$ values for the Farmer-type ionization chambers used in the institutions are reasonable and reflect the actual conditions in the settings for reference dosimetry. This alignment between the theoretical estimations and practical measurements underscores the reliability of the ${k}_{Q,{}^{60}\mathrm{Co}}$ values in the context of photon reference dosimetry.

For the electron beams, the differences in the calibration coefficients of the ionization chamber had a greater impact on this audit results. First, the use of ${N}_{D,\mathrm{w},Q}$ instead of ${N}_{D,\mathrm{w},{}^{60}\mathrm{Co}}$ in this audit improved the mean differences in the agreements from 1.3% to 0.8%, as shown in Fig. 4. This suggests that there may be a systematic error in the ${k}_{Q,{}^{60}\mathrm{Co}}$ values [33] of the Farmer-type ionization chamber used in this audit for the electron beams. Furthermore, when institutions used the cross-calibration coefficients instead of the direct-calibration coefficients, the mean differences of the agreements improved from 0.8% to −0.1%, as shown in Fig. 5. This suggests two possibilities. One is there may be a systematic error in the ${k}_{Q,{}^{60}\mathrm{Co}}$ values [11] of the parallel-plate ionization chambers used by institutions for electron beams. In the JSMP12 protocol used in the institutions, the perturbation correction factor for the air cavity (*P*_cav_) of ^60^Co γ-ray in the ${k}_{Q,{}^{60}\mathrm{Co}}$ is assumed to be 1.000. However, several studies suggested that *P*_cav_ was 1.008 for PTW Roos and 1.006 for IBA NACP-02 [35, 36]. If these *P*_cav_ values were considered for the ${k}_{Q,{}^{60}\mathrm{Co}}$ values for the institutional measured dose, the differences between the measured dose in this audit and institutions could be improved. The other possibility may be the influence of an ionization chamber holder in the institutions. A Farmer-type ionization chamber has a stem, ideally allowing the ionization chamber cavity to be placed at a reference point in the water phantom. On the other hand, a parallel-plate ionization chamber is placed at the reference point with the holder. Therefore, the holders may be causing perturbations that are not taken into account in ${k}_{Q,{}^{60}\mathrm{Co}}$ and may be affecting the measured dose. The influence of an ionization chamber holder could be canceled out by cross calibration, which may be one possible reason for the excellent agreement of this dosimetry audit results with institution cross-calibrated parallel-plate ionization chambers. Based on these results, it was decided to use ${N}_{D,\mathrm{w},Q}$ in this dosimetry audit. It has also been suggested that considering cross-calibration coefficients for electron reference dosimetry may be a reasonable option for institutions.

Another significant advantage of using the calibration coefficients determined using linac beams is their potential to reduce the uncertainties associated with determining the dose to water. As shown in Tables 3 and 4, ${k}_{Q,{}^{60}\mathrm{Co}}$ was the primary source of uncertainty for the measured absorbed dose to water of the photon beams. ${N}_{D,w,Q}$ can eliminate the need of ${k}_{Q,{}^{60}\mathrm{Co}}$ in determining the measured absorbed dose to water, thereby substantially reducing the relative standard uncertainty in this audit. Furthermore, the use of ${N}_{D,w,Q}$ in this audit enabled the evaluation of uncertainties in institutional reference dosimetry, including individual variations in ionization chamber-specific ${k}_{Q,{}^{60}\mathrm{Co}}$ values. For the uncertainties of electron beams as shown in Table 5, the relative standard uncertainty with the calibration in linac beams was larger than those with calibration in ^60^Co γ-ray. This can be attributed to the fact that the uncertainty in ${k}_{Q,{}^{60}\mathrm{Co}}$ was estimated to be as low as 0.2%. It is important to note that the ${k}_{Q,{}^{60}\mathrm{Co}}$ value was derived using a Monte Carlo simulation. These associated uncertainties were primarily considered from two aspects: (i) the statistical uncertainty inherent in the Monte Carlo simulation and (ii) the uncertainty owing to fitting variations in the beam quality. Given these considerations and the results of this audit, as shown in Figs 4 and 5, it is conceivable that the actual uncertainty in ${k}_{Q,{}^{60}\mathrm{Co}}$ for electron beams might be somewhat larger than that currently estimated.

Regarding limitations of this audit, first is that this audit used common dosimetry parameters for each beam quality, as shown in Tables 1 and 2. However, this impact could be considered minimal because the SDs of the agreements were quite small (±0.4% for photon and ±0.8% for electron reference dosimetry). These results indicate that this dosimetry audit may not be required to measure the dosimetric parameters at each institution. Second limitation is the 6 MeV electron beam evaluation in this audit. One of these limitations is the depth of measurement, which is 1 cm for a 6 MeV electron beam. *PDD*(d_m_) for 6 MeV was 0.958, which differs from the value of 1.000. It includes other uncertainties, such as the determination of the effective point of measurement (EPOM) and the selection of detectors. The EPOM of the microDiamond detector was set as the *R*_50_ value of microDiamond detector which is equal to the *R*_50_ value determined from the *PDD*s measured using Roos and Advanced Markus for each electron energy. The EPOM of the plane-parallel chambers displaced the water-equivalent entrance window thickness from the surface. Recently, the EPOM of plane-parallel chambers was reported to be the optimum value from Monte Carlo simulations [8]. Another limitation of 6 MeV is that ${N}_{D,\mathrm{w},Q}$ for a 6 MeV electron beam was obtained by extrapolation from ${N}_{D,\mathrm{w},Q}$ given at 9 and 12 MeV in the PSDL. Although there were limitations in this audit, a good agreement was observed for all energy of both photon and electron.

As shown in Fig. 4, the IBA PPC40 chamber exhibits larger systematic differences than the other plane-parallel chambers. One reason for this would be the inappropriate ${k}_{Q,{}^{60}\mathrm{Co}}$ used at the institutions. Because ${k}_{Q,{}^{60}\mathrm{Co}}$ of IBA PPC40 was not listed in the dosimetry protocol of JSMP12 [11], institutions using IBA PPC40 for electron reference dosimetry adopted ${k}_{Q,{}^{60}\mathrm{Co}}$ of PTW Roos, which have nearly identical outer and inner dimensions. From our results as shown in Fig. 5, it could be considered that the institutions having IBA PPC40 should not use it for electron reference dosimetry with the direct-calibration coefficient. Furthermore, even if the ${k}_{Q,{}^{60}\mathrm{Co}}$ for the IBA PPC40 was listed in the JSMP12 [11], it would not be certain the systematic differences could be solved. Muir *et al.* [37] revealed the differences of the ${k}_{Q,{}^{60}\mathrm{Co}}$ between Monte Carlo simulation and the water calorimeter measurement, as −1.2% for IBA PPC-40.

Based on the uncertainties and results of this audit, the tolerance limit was determined as ±1.5% for photon beams, and ±2.0% for electron beams. As mentioned in the Introduction, setting this tighter tolerance could be considered important for audits. This is because the tolerance in this audit is consistent with the IAEA’s recommendations of 1–2% for systematic bias of the reference dose [1, 2].

## CONCLUSION

An on-site dosimetry audit using the calibration coefficients of the absorbed dose to water for the ionization chamber with high-energy photon and electron beams from the linac was established with tighter tolerance, and its usefulness was demonstrated. For photon beams, the dosimetric impact of introducing the calibration coefficients determined using linac beams instead of ^60^Co γ-ray for a PTW 30013 Farmer chamber was small. For electron beams, the dosimetric impact was larger, and the measured doses in this audit using ${N}_{D,\mathrm{w},Q}$ were the most consistent with the institution-measured dose, which was evaluated using cross-calibration coefficients. Therefore, it is recommended to adopt a cross-calibration coefficient for electron reference dosimetry in institutions. Hopefully, this audit will help institutions determine how to calibrate their ionization chambers for reference dosimetry and contribute to medical safety.
